# Is It Mine or Not Mine? Cognitive Offloading to Generative AI and Concern over the Erosion of Cognitive Ownership

**DOI:** 10.3390/jintelligence14080182

**Published:** 2026-08-07

**Authors:** Jing Su, Zhuo Wang, Zhen Qiang, Zhonghou Wang

**Affiliations:** 1 School of Educational Sciences, Qingdao University, Qingdao 266071, China; 2 College English Department, School of Foreign Languages, Lanzhou Jiaotong University, Lanzhou 730070, China

**Keywords:** cognitive offloading, agency, cognitive ownership, metacognition, generative AI, GenAI dependency

## Abstract

Cognitive offloading—delegating cognitive work to external tools—is basic to human cognition, but generative AI (GenAI) amplifies it radically: entire cognitive products can now be produced on request. This sharpens a question about agency over one’s own thinking: when cognition is habitually offloaded, does its product still feel like one’s own? We surveyed 239 pre-service teachers, operationalizing the felt loss of cognitive ownership as concern over the erosion of teaching-design subjectivity (TSC)—the metacognitive appraisal that AI-assisted work is not genuinely one’s own and that independent capacity is declining. Guided by the Interaction of Person-Affect-Cognition-Execution (I-PACE) model, we tested whether AI anxiety/fear of missing out (affective) and impulsivity (self-regulatory) relate to this concern through behavioral GenAI dependency—habitual offloading. In a structural equation model with bias-corrected bootstrapping, dependency strongly predicted TSC and partially mediated the effect of AI anxiety; impulsivity raised dependency but showed a suppression pattern (a positive indirect effect offset by a null total effect), and the model explained 35% of the variance in the concern. Habitual offloading—that is, GenAI dependency—rather than generic AI use, accompanies the metacognitive loss of cognitive ownership.

## 1. Introduction

Human cognition has always been distributed: people offload memory and computation onto notebooks, calculators, and one another, trading internal effort for external support (Risko & Gilbert, 2016). Generative artificial intelligence (GenAI) marks a step change in this long history. Where earlier tools stored or retrieved the products of thought, GenAI produces them—drafting arguments, solving problems, and designing complex artifacts on request—so that the cognitive work itself, not merely its record, can be delegated (Stadler et al., 2024). This capability has been absorbed with unusual speed into education, where students now complete demanding cognitive tasks with tools such as ChatGPT (Mishra et al., 2023; Moradi, 2025). It also sharpens a question about cognition that predates AI but that AI makes acute: when the work of thinking is habitually handed to an external system, does its product still register as one’s own? This marks a categorical shift from offloading by self-documentation—writing a note that one must still encode, retrieve, and later reintegrate oneself—to offloading by machine, in which the generative act itself is performed by the system; it is the latter that raises the question of ownership we address here.

That question is, at bottom, one about the sense of agency—the experience of being the author of one’s own mental and behavioral acts (Haggard, 2017). A person who completes a complex cognitive task through AI may be unable to answer a simple but consequential question about the result: is it mine, or not mine? The question has two facets the construct is designed to capture. One is a doubt about ownership and authenticity—whether the product, and the competence it appears to display, genuinely issues from oneself. The other is a worry about decline—whether one’s independent capacity to perform the task is atrophying through reliance, consistent with evidence that offloading can trade immediate performance against durable internalization (Sparrow et al., 2011). We study this metacognitive concern in a domain where complex cognition is both central and consequential: the design of instruction by pre-service teachers, whose developing competence is precisely what habitual offloading might hollow out. In that domain, we name the felt loss of cognitive ownership “concern over the erosion of teaching-design subjectivity”, and we are explicit that it is the person’s own metacognitive appraisal of possible erosion, not a measure of the underlying capacity and not an observed decline in it. Cognitive ownership in this psychological sense—the first-person experience of authorship—is distinct from legal or intellectual-property ownership of the artifact: a design may be one’s property yet not feel like one’s own, and it is this experiential sense, not the legal one, that we study. Put plainly, the concern is the worry that one is becoming less the author of one’s own designs and more a conduit for the tool.

The concern matters for how we understand cognition in AI-augmented environments. If the felt ownership of one’s cognitive products can come apart from one’s actual reliance on external systems, then an account of intelligence under such conditions must include not only what a person can do, but whether they experience what they do as their own—and what makes that experience erode. The self-appraisals formed while acquiring a complex skill are, moreover, durable and are carried into subsequent practice (Lamote & Engels, 2010); a learner who doubts the authorship of his or her own work begins from a position of vulnerability rather than agency (Lasky, 2005). Yet the conditions that give rise to this metacognitive concern, and the mechanism through which they operate, remain unclear. Research has clarified why learners adopt GenAI—applying established technology-acceptance models (Davis, 1989) in recent studies of GenAI uptake (Moradi, 2025; Yang & Appleget, 2024)—and has begun to document problematic reliance and its cognitive costs (Stadler et al., 2024), but it has not asked what shapes whether people come to doubt the ownership of their own cognitive work. The present study addresses this gap. It examines how an affective condition (AI anxiety together with fear of missing out) and a self-regulatory disposition (impulsivity) relate to the concern, and whether behavioral GenAI dependency—habitual cognitive offloading—is the mechanism that links them.

## 2. Literature Review and Hypotheses

This section first clarifies what subjectivity means and how teaching-design subjectivity relates to, but differs from, teacher agency, and it states plainly that the concern construct is newly introduced here. It then considers how GenAI reliance may give rise to that concern, identifies the affective and dispositional conditions that drive reliance, and on this basis develops four hypotheses.

### 2.1. Subjectivity, Teacher Agency, and a New Construct

The term subjectivity carries two meanings that must be separated. In everyday, epistemic usage, it denotes reliance on personal feeling or opinion rather than external fact—the sense in which a judgment is “subjective” rather than “objective,” and in which GenAI outputs may themselves be called subjective when their training data and algorithms are not neutral. We do not use the term in that sense. We use it in its philosophical sense, in which a subject is an agent who authors his or her own action and experience rather than an object to whom action is done; teaching-design subjectivity, so understood, is the teacher’s standing as the author and owner of a particular instructional design. To avoid the epistemic confusion, we anchor the construct throughout in the language of cognitive ownership and authorship rather than of subjectivity versus objectivity. Carried into education, the idea concerns the degree to which a teacher is the originating author of his or her pedagogical decisions. This idea is closely related to teacher agency, which the teacher-education literature defines as the capacity to act intentionally and to exercise judgment over one’s own work and professional formation (Vähäsantanen, 2015). This capacity is widely theorized as subject-centred and resourced—exercised by an agent who draws on personal and contextual resources (Eteläpelto et al., 2013)—and as conditioned by the teacher’s own beliefs and the ecological circumstances of practice (Biesta et al., 2015). Teacher agency, however, names a capacity—what a teacher is able to do. Teaching-design subjectivity, as we use it, names a narrower and more experiential matter: the teacher’s standing as the author and owner of a particular instructional design, and the first-person sense that the design issues from the self. A teacher may possess design agency in general yet, on a given AI-assisted task, experience the design as not genuinely his or her own. It is this experiential, ownership-centred aspect, rather than agency as a capacity, that GenAI most directly unsettles, and that the present study addresses.

We make two commitments explicit. First, neither teaching-design subjectivity nor the concern about its erosion is an established, validated construct in the literature; both are introduced here, and we therefore accept the corresponding burden of construct definition and validation, addressed in the Method. Second, the variable we measure is the concern, not the subjectivity. Measuring subjectivity itself would require an index of design capacity, and measuring its erosion would require repeated measurement or an objective performance criterion; we have neither. What we can measure, and what is professionally consequential in its own right, is the pre-service teacher’s concern that, under reliance on AI, the design is no longer his or her own and the underlying capacity is declining. This concern is the dependent variable throughout. We stress that this is the person’s subjective appraisal of ownership, which need not track the objective facts of authorship: heavy reliance may, objectively, mean a product is not largely one’s own, yet the felt sense of ownership can run ahead of or behind that reality, and it is precisely this potential divergence that we study. How closely the appraisal tracks reality may itself depend on the user’s knowledge—someone who knows a subject well, or who understands how AI generates its output, may better judge both its quality and their own contribution—so domain knowledge is a plausible moderator for future work. The concern is also bounded against two neighbouring constructs. Unlike imposter syndrome—a generalized, trait-like fear of being exposed as a fraud across achievement domains (Clance & Imes, 1978)—it is tied specifically to the authorship of AI-assisted work and to an identifiable behavioral cause; and unlike AI anxiety—an affective apprehension about the technology and one’s standing relative to it—it is a metacognitive appraisal of ownership, and the two prove empirically separable (HTMT = 0.44; Section 5.1).

For pre-service teachers in particular, such concern is developmentally significant, because the consolidation of a professional identity—including the conviction that one authors one’s own pedagogical decisions—is a central task of the preparation period (Wong & Liu, 2022). Concern over the erosion of that authorship therefore speaks to a formative process, not merely to a passing worry.

### 2.2. Generative AI Reliance and Concern over Erosion

How might GenAI use give rise to this concern? The mechanism we propose is behavioral over-reliance, understood through the cognitive-offloading account, in which the transfer of effortful processing to an external aid is adaptive when selective but detrimental when it becomes habitual and indiscriminate, since it displaces the self-regulated cognition through which competence is built (Risko & Gilbert, 2016) and forgoes the effortful, germane processing through which durable learning occurs (Kirschner, 2002). These concerns are not merely theoretical: cognitive offloading reshapes what people retain and how they reason (Gilbert, 2024), heavier reliance on AI has been associated with weaker critical thinking (Gerlich, 2025), and research on human–automation interaction documents a parallel tendency toward complacency and the uncritical acceptance of a system’s output (Wickens et al., 2015), including overreliance on AI in assisted decision-making (Buçinca et al., 2021). Repeated, undiscriminating offloading is consequential because it can reduce the mental effort invested in a task (Stadler et al., 2024). Moreover, individuals differ in how readily they offload (Morrison & Richmond, 2020), so that some pre-service teachers come to conduct most of their instructional design through AI. A teacher whose design is routinely produced this way accumulates concrete, first-person experience of not exercising independent judgment, and it is plausibly this experience—rather than mere exposure to the technology—that prompts the question of whether the work is genuinely his or her own. Reliance is not monolithic, however: a learner may offload a routine subtask such as translation or outlining while still reasoning independently about a genuinely complex design, and learners differ in whether they construe instructional design as a routine chore or as demanding sustained thought. Our dependency measure indexes general reliance, and the outcome is domain-specific; task-level heterogeneity in what is offloaded is revisited in the limitations.

Behavioral GenAI dependency—here defined as habitual, default reliance on the tool, that is, defaulting to AI even for tasks one could perform alone, difficulty working without it, and the uncritical adoption of its outputs—thus represents the most direct behavioral route to the concern. The broader evidence on GenAI and learning is mixed: some studies report gains in self-efficacy and motivation (Shao & Wang, 2026), whereas others document procrastination and over-attachment (J. Zhang & Zhou, 2026), alongside heightened ethical concern about the authorship of AI-assisted work (Uppal & Hajian, 2024). This divergence suggests that consequences depend on how use is enacted, and it directs attention to dependency as the proximal behavioral condition most likely to provoke doubt about ownership. The phenomenon is concrete for those who design and revise instructional materials through AI (Koltovskaia et al., 2024), for whom the authenticity of the resulting competence is an immediate professional question.

### 2.3. Affective and Dispositional Drivers of Dependency

If dependency is the proximal mechanism, the question becomes what drives it. Two classes of antecedent—distinct from each other, and not intended as exhaustive—are well established in research on problematic technology use; others, such as time pressure, may also drive reliance but lie beyond the present model. The first is affective. Fear of missing out—the apprehension that others are benefiting from experiences from which one is absent—is consistently associated with compulsive technology engagement (Przybylski et al., 2013), and an analogous AI-related anxiety has emerged among students who fear being disadvantaged relative to more AI-fluent peers; such anxiety can be construed as an achievement-related emotion that arises from appraisals of limited control under high stakes (Pekrun, 2024). What makes such anxiety a driver of dependency is its regulatory logic: anxiety-driven engagement is governed by the relief of an aversive state rather than by appraisal of the task, a mechanism shown to foster problematic technology use (Elhai et al., 2016) and consistently tied to its depressive and anxious correlates (Elhai et al., 2020). Engagement of this kind tends to become habitual and undiscriminating, which is the defining feature of dependency. Hence:

**H1.**  *AI anxiety/fear of missing out is positively associated with behavioral GenAI dependency.*

The second class of antecedent is dispositional. Trait impulsivity—a preference for immediate reward over effortful, delayed payoff, as captured by the Barratt Impulsiveness Scale (Patton et al., 1995)—is a disposition widely theorized to underlie problematic technology use. Because deferring gratification is a foundational self-regulatory resource (Mischel et al., 1989), individuals lower in it tend to discount the deferred, diffuse costs of offloading relative to its immediate rewards, which makes the instantaneous outputs of GenAI difficult to resist. Hence:

**H2.**  *Impulsivity is positively associated with behavioral GenAI dependency.*

A related dispositional resource is academic self-efficacy. Learners who doubt their capacity to succeed unaided may turn to AI defensively, so that low self-efficacy can itself feed dependency (Bandura, 1977); we introduce it here because it recurs in the implications, where strengthening self-efficacy is proposed as one lever against defensive reliance.

### 2.4. Dependency as the Mediating Mechanism

The argument implies a specific structure. AI anxiety and impulsivity are general states that are not, in themselves, about teaching; their relevance to a pre-service teacher’s doubt about the ownership of instructional work is realized through behavior. The two conditions are expected to elevate behavioral dependency, and the accumulated experience of designing through AI is, in turn, expected to heighten concern over the erosion of teaching-design subjectivity. Dependency thus occupies the mediating position between the antecedents and the concern:

**H3.**  *Behavioral GenAI dependency is positively associated with concern over the erosion of teaching-design subjectivity.*

**H4.**  *Behavioral GenAI dependency mediates the associations of (a) AI anxiety/fear of missing out and (b) impulsivity with concern over the erosion of teaching-design subjectivity.*

No existing body of work is positioned to evaluate this account. Research on technology adoption explains why students begin to use these tools (Venkatesh et al., 2003; Moradi, 2025) but not what reliance does to their sense of ownership over their work; research on AI dependence has examined its antecedents and cognitive correlates (S. Zhang et al., 2024) and has begun to position it as a mediator of academic outcomes such as procrastination (J. Zhang & Zhou, 2026), yet without connecting it to teaching; and research on teacher agency has theorized professional subjectivity without reference to the pressures introduced by GenAI (Tao & Gao, 2017). The present study joins these strands.

## 3. Theoretical Framework

To organize these hypotheses within a single explanatory structure, the study adopts the Interaction of Person-Affect-Cognition-Execution (I-PACE) model, a leading account of how problematic technology-use patterns develop and are maintained (Brand et al., 2014). I-PACE holds that problematic use arises from the interaction of relatively stable person characteristics with affective and cognitive responses to relevant triggers, which together shape the executive behavior of using—and over-using—a technology, with consequences that feed back into subsequent use (Brand et al., 2016). Its logic extends naturally to GenAI, a technology that is instantly available, effortlessly rewarding, and increasingly integral to academic work.

**Figure 1:**
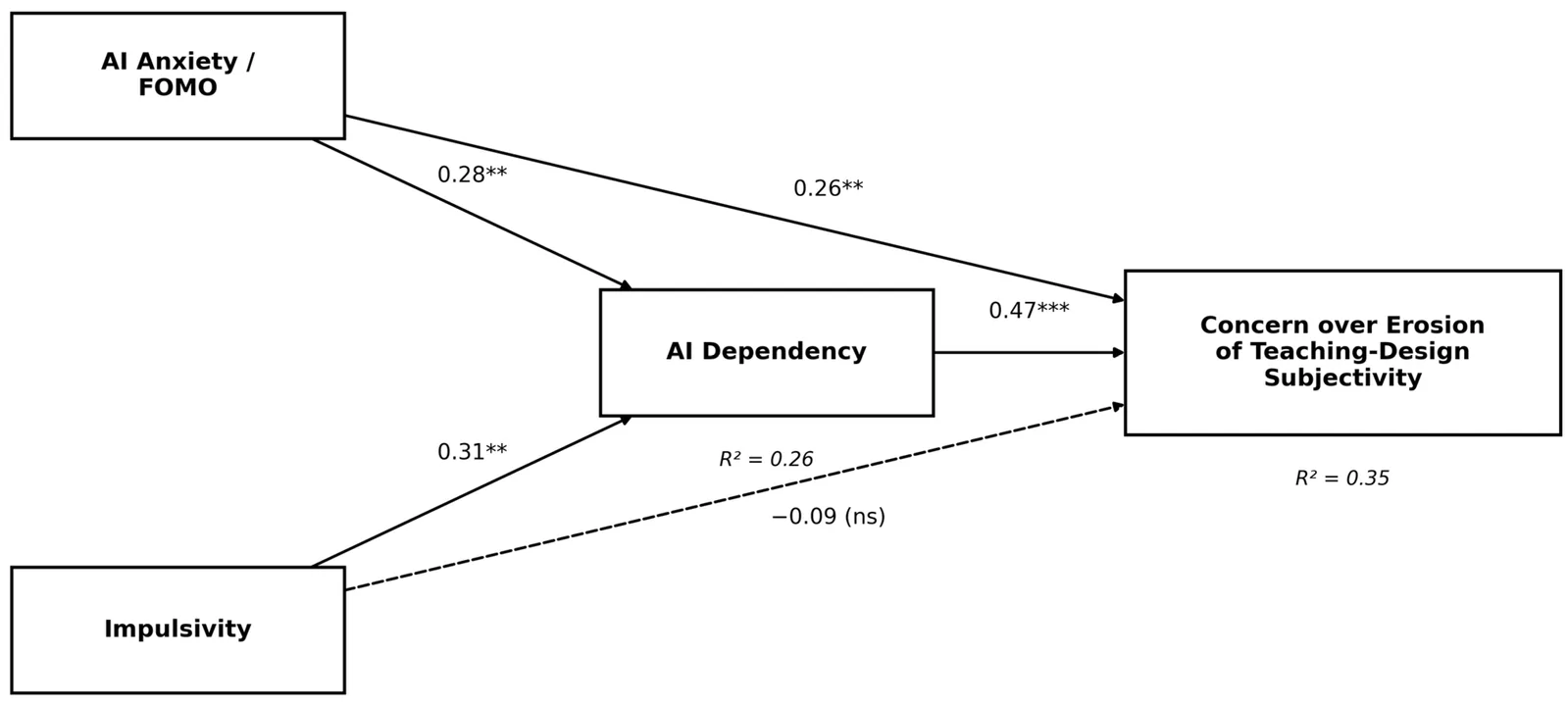
Structural Model With Standardized Path Coefficients. Note. Solid arrows = significant paths; dashed grey arrows = non-significant direct effects. ** *p* < .01, *** *p* < .001.

Mapping the present constructs onto this structure, impulsivity is the predisposing person factor—a lapse of self-regulatory control that spans both the “cool” cognitive and the “hot” affective components of executive function (Metcalfe & Mischel, 1999; Zelazo & Carlson, 2012)—AI anxiety and fear of missing out the affective response, and behavioral GenAI dependency—habitual cognitive offloading—the executive outcome. The concern over the erosion of teaching-design subjectivity is positioned as a consequence of that executive behavior; specifically, a negative metacognitive appraisal—a cognition about one’s own cognition rather than an emotion about the self—of one’s own dependent conduct. It is a judgment about the ownership and trajectory of one’s own cognition, of the kind I-PACE locates in the feedback loop through which the experience of use shapes the user. We are careful not to over-read this consequence: it is an appraisal the person forms about the ownership and trajectory of his or her competence, not a change in identity, which we do not measure. So specified, I-PACE supplies the rationale for the hypothesized ordering in which behavior mediates the influence of affect and disposition on the concern, and it is this framework that we carry through the interpretation of the results. The model is depicted in Figure 1.

## 4. Method

### 4.1. Participants and Procedure

Participants were 239 pre-service teachers (education-major university students) in Eastern China, recruited by convenience sampling from a larger, voluntary web-based survey of university students’ GenAI use. The study protocol was approved by the Institutional Review Board of Qingdao University. Informed consent was obtained online: the consent statement appeared on the first screen of the questionnaire and had to be accepted before any item could be accessed. From the 264 respondents in the teacher-education track, to whom the teaching-subjectivity items were administered, we excluded those who reported never having used GenAI and a small number whose responses showed no variance across the focal items, yielding the analytic sample of 239. The sample was 92.9% female and was concentrated in the second and third years of study, and reported GenAI use ranged from monthly or less to several times per day, with most respondents using it weekly or more. Full demographic and usage characteristics are reported in Table 1.

### 4.2. Measures

All constructs were measured on seven-point Likert scales (1 = strongly disagree, 7 = strongly agree). Because concern over the erosion of teaching-design subjectivity (TSC) is a newly introduced construct without existing instruments, the authors developed a scale for them following established scale-development guidance (Clark & Watson, 2019; Boateng et al., 2018). Item development proceeded from explicit construct definitions—grounded in the teacher-agency and professional-identity literature (Vähäsantanen, 2015)—to an initial item pool written to reflect each definition, which the research team then reviewed and refined for clarity and content coverage; the full item wording is provided in the Appendix A. Other scales including AI anxiety and fear of missing out (ANX; six items), Impulsivity (IMP; two items), and Behavioral GenAI dependency (DEP; four items) were adopted from Jiang and Wang (2026).

Analyses were conducted in R using lavaan. We used structural equation modeling because it estimates the constructs as latent variables—correcting the structural paths for measurement error—and lets the mediation hypotheses be tested directly. Because the items departed only modestly from multivariate normality, the confirmatory factor analysis and the structural model were estimated with the robust maximum likelihood (MLR) estimator, and indirect effects were evaluated with bias-corrected bootstrapping using 5000 resamples (Preacher & Hayes, 2008; MacKinnon et al., 2004). Model fit was assessed against the criteria of CFI and TLI at or above 0.95 and RMSEA and SRMR at or below 0.08 (Hu & Bentler, 1999). Two within-factor residual covariances between similarly worded items were freed on the basis of modification indices and theoretical justification (Cole et al., 2007). Because all measures were self-reported, the potential for common method bias was examined (Podsakoff et al., 2003). Although seven-point Likert items are ordinal, items with five or more ordered categories, provided their thresholds are not extremely asymmetric, are recovered accurately when treated as continuous under a robust estimator, and with seven categories continuous estimation is often preferable (Rhemtulla et al., 2012; Robitzsch, 2020; C.-H. Li, 2016); because our items depart only modestly from symmetry, we therefore used MLR for the primary analysis and, as a robustness check, re-estimated every model with the ordinal WLSMV (polychoric) estimator (Section 5.3).

## 5. Results

### 5.1. Measurement Model and Discriminant Validity

The four-factor measurement model fit the data well: scaled χ^2^ = 159.77, *df* = 111, *p* = .002, CFI = 0.97, TLI = 0.97, RMSEA = 0.04 (90% CI [0.03, 0.06]), SRMR = 0.05. All standardized loadings were substantial and significant, and every construct exceeded conventional thresholds for reliability and convergent validity (composite reliability: ANX = 0.89, IMP = 0.79, DEP = 0.80, concern = 0.90; average variance extracted: ANX = 0.63, IMP = 0.65, DEP = 0.58, concern = 0.64; Table 2). Because impulsivity was measured with two items—for which Cronbach’s alpha is not the most appropriate reliability estimate (Eisinga et al., 2013)—we relied on composite reliability from the CFA.

Because the focal outcome is a form of concern, its discriminant validity from AI anxiety is the critical measurement question, and we addressed it directly. The two constructs were only moderately correlated (φ = 0.42), their heterotrait–monotrait ratio of correlations was well below the recommended 0.85 threshold (HTMT = 0.44; Henseler et al., 2015), and a model that merged the anxiety items and the concern items into a single factor fit far worse than the four-factor model (CFI dropping from 0.97 to 0.74; scaled Δχ^2^(3) = 187.64, *p* < .001). Concern over the erosion of teaching-design subjectivity is therefore empirically distinct from AI anxiety rather than a relabeling of it. Discriminant validity among the remaining constructs was likewise supported, with all HTMT ratios at or below 0.50; in particular, although several concern items reference reliance on AI, the concern remained discriminant from dependency (HTMT = 0.49). Common method bias was unlikely to be the principal explanation of the results, as Harman’s single-factor test attributed only 40.1% of the variance to one factor and a one-factor model fit poorly (CFI = 0.52); moreover, in an unmeasured-method-factor model a common method factor explained only 8.8% of item variance, far below the 53.3% explained by the substantive factors, and every substantive loading remained significant with it in the model. We nonetheless treat single-source variance as a limitation.

### 5.2. Structural Model and Mediation

The structural model is presented in Figure 1. Because every antecedent was allowed to predict both the mediator and the outcome, the structural component is just-identified (saturated); the global fit reported above therefore reflects the measurement model, and the mediation hypotheses are evaluated through the bias-corrected bootstrap indirect effects rather than through overall fit. Consistent with H1 and H2, both AI anxiety (β = 0.28, *p* = .002) and impulsivity (β = 0.31, *p* = .009) predicted behavioral GenAI dependency, together accounting for 26% of its variance. Consistent with H3, dependency strongly predicted concern over the erosion of teaching-design subjectivity (β = 0.47, *p* < .001), and the model accounted for 35% of the variance in the concern. In a saturated model, the structural paths exhaust the available degrees of freedom, so fit cannot be tested at that level and the hypotheses are judged from the estimated effects themselves.

Direct, indirect, and total effects are reported in Table 3. For clarity, the direct effect is the association between an antecedent and the concern that remains after dependency is accounted for; the indirect effect is the part transmitted through dependency (antecedent → dependency → concern); and the total effect is the sum of the two. For AI anxiety, the total effect on the concern was substantial and significant (β = 0.39, 95% CI [0.22, 0.56]); it comprised a significant indirect effect through dependency (β = 0.13, 95% CI [0.05, 0.25]) and a direct effect that was also significant (β = 0.26, *p* = .004). AI anxiety thus relates to the concern both through dependency and beyond it—a pattern of partial mediation that supports H4a. Following the contemporary emphasis on the indirect effect itself as the test of mediation rather than on the full-versus-partial dichotomy (Rucker et al., 2011), we read the robust indirect path as the substantive finding: a meaningful share of anxiety’s association with the concern is realized through dependent behavior.

For impulsivity, the picture was different and is reported transparently. Impulsivity predicted dependency (H2) and had a positive and significant indirect effect on the concern through it (β = 0.15, 95% CI [0.04, 0.28], *p* = .017), yet its total effect on the concern was essentially zero and non-significant (β = 0.06, 95% CI [−0.17, 0.28]), because the positive indirect effect was offset by a negative, non-significant direct path (β = −0.09). This is a pattern of inconsistent (competitive) mediation, or suppression, in which a genuine indirect effect coexists with a null total effect (MacKinnon et al., 2000). H4b is therefore supported at the level of the indirect effect—impulsivity does reach the concern through dependency—but we emphasize the suppression: because impulsivity has no net association with the concern, its role is confined to the behavioral pathway and should not be read as an overall risk factor for the concern itself.

### 5.3. Robustness Checks

Four checks supported the reported model. First, the mediation was re-estimated with an item-parceled latent model (Little et al., 2002) and with an observed-composite model; both indirect effects retained their sign and significance, and the impulsivity suppression pattern—a positive indirect effect coexisting with a null total effect—likewise recurred, indicating that the results are not an artifact of one measurement approach. Second, the path from dependency to the concern remained strong and significant (β = 0.51, *p* < .001) after the inclusion of year of study, gender, use frequency, and payment status as covariates, none of which materially altered the structural estimates. Third, a Monte Carlo estimate of power for the AI-anxiety indirect effect was approximately 0.93. Fourth, because confirming a newly developed measure in the same sample used to test hypotheses risks capitalizing on chance, the measurement model was cross-validated on random split-halves: an exploratory factor analysis on one half recovered the four intended factors with clean simple structure (no cross-loadings above 0.40), the a priori four-factor model fit the held-out half well (CFI = 0.96, RMSEA = 0.06), and the model exhibited full scalar invariance across the two halves (ΔCFI = 0.00 for both the loading and the intercept constraints); the qualitative structural pattern—a strong dependency-to-concern path, a significant direct anxiety effect, and the impulsivity suppression—recurred in each half. The full-indicator latent model is reported as the primary specification: at the present sample size, it yielded a stable bootstrap distribution, with fewer than 1% of resamples (0.8%) returning non-admissible solutions, so that parceling was unnecessary and is retained only as one of the robustness checks above. Fifth, because the items are ordinal, all models were re-estimated with the WLSMV (polychoric) estimator (Flora & Curran, 2004; C.-H. Li, 2016): the measurement model continued to fit well (CFI = 0.98, TLI = 0.97) and every structural conclusion was reproduced—both antecedents predicted dependency (AI anxiety β = 0.23, impulsivity β = 0.35, both *p* < .001), dependency predicted the concern (β = 0.50, *p* < .001), AI anxiety retained a significant direct path (β = 0.33, *p* < .001), and impulsivity again showed suppression, a significant positive indirect effect (β = 0.17, *p* < .001) coexisting with a null total effect (β = 0.02, ns). The ordinal treatment thus left every substantive conclusion intact; the one qualitative change was that impulsivity’s direct path, non-significant under MLR (β = −0.09), reached significance under WLSMV (β = −0.15), which strengthens rather than weakens the suppression pattern (Appendix A Table A2).

## 6. Discussion

This study introduced concern over the erosion of teaching-design subjectivity—the pre-service teacher’s metacognitive self-questioning about whether AI-assisted instructional work is genuinely his or her own and whether independent design capacity is declining—and asked what gives rise to it. The hypothesized model was supported. AI anxiety (H1) and impulsivity (H2) both predicted behavioral GenAI dependency; dependency strongly predicted the concern (H3); and dependency conveyed the influence of both antecedents on the concern (H4a, H4b). For AI anxiety, the mediation was partial—dependency carried part of the effect while a direct association remained—whereas for impulsivity, the mediation was of the suppression type: a genuine positive indirect effect through dependency coexisted with a null total effect, a pattern we return to below.

The principal contribution is to specify, within the I-PACE framework, how an affective condition reaches a professional appraisal by way of behavior. I-PACE locates problematic use at the executive stage and posits a feedback loop in which the experience of use shapes the user (Brand et al., 2016); the present results give that loop a concrete, education-specific content. The executive behavior of AI dependency does not merely persist or recur—it becomes the object of a negative self-appraisal, the doubt about ownership that the title of this article names. Anxiety reaches the concern by two routes: partly through the dependent behavior it fosters—whose meaning the teacher then reads—and partly through a direct route that likely reflects the diffuse apprehension anxious students carry into appraisals of their own competence. That the indirect route is robust across specifications is the more precise, education-specific claim, going beyond the general proposition that AI use erodes competence (Stadler et al., 2024): it identifies the behavioral mechanism, not merely the affective correlate, through which the worry is realized.

The result invites comparison with recent studies that have placed AI reliance in a mediating role. J. Zhang and Zhou (2026) found that generalized AI reliance was associated with academic procrastination through reduced academic self-efficacy and self-regulated learning; the present study converges with their placement of reliance as a mechanism but differs in outcome, showing that reliance reaches not only performance-related outcomes such as procrastination but a teacher-specific appraisal of ownership. H. Li et al. (2026) found cognitive load and future anxiety to mediate the link between task complexity and AI dependency; our findings extend this line by demonstrating that the downstream appraisal can concern the authorship of professional work, not only affect or effort. Where these studies treat dependency largely as a conduit to generic academic outcomes, the present study shows that, for a professionally oriented population, the conduit terminates in a question about the self as author. Related work has examined AI-chatbot dependence in relation to loneliness and perceived social support in young adults (Uğuz et al., 2026) and has begun to treat affective dependence on GenAI as a measurable construct among students (Yao et al., 2026); the present study differs in carrying the dependency mechanism into a specifically teacher-related appraisal of design ownership.

The impulsivity finding deserves equal emphasis, precisely because it did not behave as a simple risk factor. Impulsive pre-service teachers were more dependent on AI, and that dependency did translate into greater concern—the indirect path was positive and significant—yet impulsivity had no overall relationship with concern about erosion, because this positive indirect path was cancelled by a negative direct path. One plausible reading is that impulsive individuals both offload more and worry about it less—their disposition simultaneously raises dependency and dampens the reflective concern that dependency would otherwise provoke. This interpretation is speculative and requires direct test, but it cautions against assuming that the dispositions which increase reliance are the same ones that increase awareness of its costs.

## 7. Implications for Cognition in AI-Augmented Environments

By the metacognitive loss of cognitive ownership we mean the person’s own second-order judgment about their first-order thinking—specifically, the appraisal that an AI-assisted product is no longer genuinely theirs; it is metacognitive because it is a judgment about one’s own cognition, not an emotion about the self. Framed in I-PACE terms, the results locate where this loss can be addressed. Because the concern is reached through the executive stage—behavioral dependency, that is, habitual cognitive offloading—rather than directly from affect or disposition, intervention at that stage is likely to be the most consequential: reducing indiscriminate offloading should attenuate both the dependency and the doubt it engenders. For learning environments generally, this favors cultivating the metacognitive regulation of when to offload and when to think unaided—a critical, evaluative stance toward AI (Long & Magerko, 2020) for which validated competency measures now exist (Carolus et al., 2023)—over the blunt reflex of restricting access, which constrains use without building the competence that keeps reliance deliberate. In the applied case that motivated this study, teacher preparation, program-level evidence indicates that thoughtfully designed AI components can strengthen rather than displace pre-service teachers’ competencies (Lee & Bryan, 2025), that learners’ own perceptions shape how they take up these tools (Yang & Appleget, 2024), and that immersive AI-based pedagogies can be embedded without supplanting professional judgment (N. Zhang et al., 2026). The present results specify the psychological target such provision should address: not GenAI use as such, but the dependency—the habitual offloading—that develops when anxious learners offload to relieve anxiety.

Two I-PACE components beyond the executive stage suggest complementary measures. At the affective stage, support for regulating AI-related anxiety may reduce the affective pressure toward offloading; at the person stage—the predisposing dispositional level of I-PACE, where impulsivity resides—the metacognitive self-regulation that counteracts impulsive offloading can be strengthened directly (Zimmerman, 1990), as can the self-efficacy that may reduce defensive reliance (Bandura, 1977). Concretely, coursework might require learners to verify and improve AI-generated material, to reflect on when delegation undermines the ownership of their own work, and—in the teacher-preparation case—to rehearse strategies for guiding future pupils toward a comparably critical stance, treating the metacognitive regulation of offloading not as a technical add-on but as a constituent of the cognitive ownership that expert performance depends on. One further implication—beyond what cross-sectional data can establish—is a developmental sequencing in which novices first build core instructional-design competence unaided and take up AI only progressively, accompanied by explicit discussion of when delegation is and is not appropriate; this scaffold-and-fade logic complements rather than contradicts the emphasis on metacognitive regulation. The construct has practical value in its own right: because it is an early, self-reported signal that appears before any objective decline can be measured, it offers teacher educators a usable early-warning indicator, and because it is reached through dependency, it points to a modifiable behavioral target rather than a fixed trait.

## 8. Limitations and Future Research

Several limitations qualify these conclusions. The most consequential concerns the cross-sectional design. Although the hypothesized model fit well, cross-sectional data cannot establish the direction of mediation; a reverse model and a non-mediation model are statistically equivalent to the hypothesized one by virtue of the saturated structural form and cannot be adjudicated empirically (Maxwell & Cole, 2007), a bias that persists even under autoregressive assumptions (Maxwell et al., 2011). The causal ordering adopted here rests on theory—on I-PACE and on the temporal logic by which a behavior precedes the appraisal of that behavior. We emphasize, however, that non-experimental cross-sectional data cannot by themselves establish mediation, and, following Weems (2025), we interpret the indirect paths associationally rather than as demonstrated causal mediation; only longitudinal or experimental designs can confirm it. Second, the focal construct and its scale are newly introduced; although their reliability, convergent validity, and discriminant validity from AI anxiety were satisfactory and the measurement model cross-validated across random split-halves with full scalar invariance, confirmation in a fully independent sample and across cultural and disciplinary contexts remains desirable. Third, the sample was 92.9% female and drawn from education majors in one regional context, which constrains generalizability and precludes a meaningful test of gender invariance. Fourth, the outcome is a self-reported appraisal and is therefore inherently subjective; this is appropriate—the construct is, by definition, a first-person appraisal—but it means the measure indexes felt rather than demonstrated erosion. Fifth, reliance may be task- or domain-specific rather than global, so that over-reliance on AI for one kind of task need not generalize to others; measuring reliance at the task level is a priority. Finally, because the sample is exclusively pre-service, we cannot compare these teachers with in-service veterans, for whom the dynamics may differ: a veteran who has consolidated design expertise may experience offloading as the loss of an existing skill, whereas a novice may never build the skill and so may not register a loss; future work should also model AI literacy and self-regulation as moderators of the paths from affect and disposition to dependency.

## 9. Conclusions

As generative AI becomes a routine instrument for accomplishing complex cognitive work, those who use it may increasingly confront a disquieting question about the results: is it mine, or not mine? This study framed that question as one about the sense of agency over one’s own cognition, introduced a measurable metacognitive construct for it in a high-stakes domain—concern over the erosion of teaching-design subjectivity—and showed, within the I-PACE framework, that a meaningful share of it arises through the behavioral dependency, the habitual cognitive offloading, that AI anxiety and impulsivity engender, with dependency accounting, alongside its antecedents, for over a third of the variance in the concern. By locating habitual offloading as the mechanism, the study indicates where the felt ownership of one’s cognitive products is won or lost. This also marks a boundary on where offloading is benign: AI is best suited to low-level, routine subtasks rather than to the complex cognition through which professional competence is built, and the risk we identify lies in habitual offloading migrating from the former to the latter.

The significance of this concern lies in what it reveals about intelligence under conditions of pervasive offloading. Accounts of what a person can do have always been framed in terms of internal capacity; but when capacity can be borrowed instantly and invisibly, a second question becomes unavoidable—whether the performer still experiences the performance as their own, and whether that experience tracks, or comes apart from, their actual reliance. The present results suggest it can come apart: a felt loss of ownership is driven not by AI use frequency, but by the habitual offloading through which reliance hardens into disposition. Because such dispositions consolidate as a skill is acquired and are carried into later performance, a doubt that takes hold now may outlast its origin and prove difficult to reverse. Whether the products of AI-augmented cognition remain, in the performer’s own eyes, genuinely theirs is thus a question the study of intelligence can now track with a defined construct and an identified mechanism—and one it can ill afford to leave unwatched.

## Figures and Tables

**Table 1:** Demographic and GenAI-Use Characteristics (N = 239).

Characteristic	Category	*n*	%
Gender	Female	222	92.9
	Male	17	7.1
Year	First year	3	1.3
	Second year	105	43.9
	Third year	117	49
	Fourth year	14	5.9
GenAI use	Daily or more	107	44.8
	Weekly	126	52.7
	Monthly or less	6	2.5
Paid for GenAI	Yes	207	86.6

Note. *n* = number of respondents; percentages are column percentages.

**Table 2:** Measurement Model: Reliability and Convergent Validity.

Construct	Items	CR	AVE
AI anxiety/FOMO (ANX)	6	0.89	0.63
Impulsivity (IMP)	2	0.79	0.65
AI dependency (DEP)	4	0.80	0.58
Teaching-subjectivity concern (TSC)	5	0.90	0.64

Note. TSC was developed for this study, while ANX, IMP and DEP were adopted from Jiang and Wang (2026). CR = composite reliability; AVE = average variance extracted. Model fit: CFI = 0.97, TLI = 0.97, RMSEA = 0.04, SRMR = 0.05.

**Table 3:** Structural Paths and Bootstrapped Indirect Effects.

**Path**	**β**	**SE**	***p***	**Sig.**
ANX → DEP (H1)	0.28	0.09	0.002	**
IMP → DEP (H2)	0.31	0.12	0.009	**
DEP → TSC (H3)	0.47	0.08	<.001	***
ANX → TSC (direct)	0.26	0.09	0.004	**
IMP → TSC (direct)	−0.09	0.10	0.398	ns
**Effect**	**β**	**95% CI (BCa)**		**Inference**
Indirect: ANX → DEP → TSC (H4a)	0.13	[0.05, 0.25]		excludes 0
Indirect: IMP → DEP → TSC (H4b)	0.15	[0.04, 0.28]		excludes 0
Total: ANX → TSC	0.39	[0.22, 0.56]		excludes 0
Total: IMP → TSC	0.06	[−0.17, 0.28]		includes 0 (ns)

Note. ** *p* < .01, *** *p* < .001; β = standardized coefficient; bias-corrected 95% bootstrap CIs (5000 resamples); an effect is significant when its CI excludes zero. R^2^ = 0.26 for DEP and 0.35 for the concern outcome. For impulsivity, a significant positive indirect effect coexists with a null total effect (suppression); H4b is supported only at the level of the indirect effect.

## Data Availability

The raw data supporting the conclusions of this article will be made available by the corresponding authors upon reasonable request.

## Appendix A. Measurement Items for Concern over the Erosion of Teaching-Design Subjectivity (TSC)

Concern over the erosion of teaching-design subjectivity (TSC). (1) I worry that long-term reliance on AI for lesson preparation will degrade my own instructional-design ability. (2) After completing teaching preparation with AI, I doubt whether it counts as my “real” professional competence. (3) I worry that, without AI, my ability to independently design lessons and solve teaching problems will decline. (4) I worry that, as a future teacher, I will over-rely on AI and lose the subjectivity a teacher ought to have. (5) I hope to reduce my reliance on AI, so as not to let my basic teaching skills atrophy.

**Table A1:** Mapping of Teaching-Subjectivity Concern (TSC) Items to Theoretical Facets.

Item	Abbreviated Wording	Facet
TSC1	… long-term reliance on AI will degrade my own instructional-design ability	Worry about decline
TSC2	… I doubt whether AI-assisted work counts as my “real” professional competence	Doubt about authenticity/ownership
TSC3	… without AI, my ability to independently design lessons will decline	Worry about decline
TSC4	… I will over-rely on AI and lose the subjectivity a teacher ought to have	Doubt about authenticity/ownership
TSC5	… I hope to reduce reliance so my basic teaching skills do not atrophy	Worry about decline

**Table A2:** Ordinal (WLSMV) Robustness Check

Path/Effect	β (WLSMV)	95% CI	β (MLR, Primary)
ANX → DEP	0.23	[0.11, 0.35]	0.28
IMP → DEP	0.35	[0.23, 0.46]	0.31
DEP → TSC	0.50	[0.41, 0.59]	0.47
ANX → TSC (direct)	0.33	[0.22, 0.43]	0.26
IMP → TSC (direct)	−0.15	[−0.27, −0.04]	−0.09
Indirect: ANX → DEP → TSC	0.12	[0.06, 0.18]	0.13
Indirect: IMP → DEP → TSC	0.17	[0.11, 0.24]	0.15
Total: ANX → TSC	0.44	[0.34, 0.55]	0.39
Total: IMP → TSC (ns)	0.02	[−0.10, 0.14]	0.06

Note. WLSMV = weighted least squares, mean- and variance-adjusted (polychoric correlations), treating all items as ordinal. The impulsivity suppression pattern—a significant positive indirect effect coexisting with a null total effect—is reproduced. Measurement model under WLSMV: CFI = 0.98, TLI = 0.97. ns: not statistically significant.
